# The role of Cobalt-60 in modern radiation therapy: Dose delivery and image guidance

**DOI:** 10.4103/0971-6203.54846

**Published:** 2009

**Authors:** L. John Schreiner, Chandra P. Joshi, Johnson Darko, Andrew Kerr, Greg Salomons, Sandeep Dhanesar

**Affiliations:** 1 Department of Medical Physics, Cancer Centre of Southeastern Ontario (CCSEO), Kingston, ON, Canada; 2 Departments of Oncology and Physics, Queen's University, Kingston, ON, Canada

**Keywords:** Cobalt-60, intensity-modulated radiation therapy, image guidance, megavoltage computed tomography, tomotherapy

## Abstract

The advances in modern radiation therapy with techniques such as intensity-modulated radiation therapy and image-guided radiation therapy (IMRT and IGRT) have been limited almost exclusively to linear accelerators. Investigations of modern Cobalt-60 (Co-60) radiation delivery in the context of IMRT and IGRT have been very sparse, and have been limited mainly to computer-modeling and treatment-planning exercises. In this paper, we report on the results of experiments using a tomotherapy benchtop apparatus attached to a conventional Co-60 unit. We show that conformal dose delivery is possible and also that Co-60 can be used as the radiation source in megavoltage computed tomography imaging. These results complement our modeling studies of Co-60 tomotherapy and provide a strong motivation for continuing development of modern Cobalt-60 treatment devices.

## Introduction

Modern radiation therapy is moving towards advanced conformal techniques such as intensity-modulated radiation therapy (IMRT) in conjunction with image guidance, bringing about image-guided radiation therapy (IGRT), to ensure accurate patient treatment. The clinical application of these advanced techniques has been limited almost exclusively to linear accelerators. Investigations of conformal Cobalt-60 (Co-60) radiation delivery have been sparse, in part because of preconceived notions that the radiation beams from Co-60 do not have the properties required in modern radiation treatment.[1] There have been a number of modeling studies that have suggested that Co-60 may be more effective in modern radiation therapy than perceived in the past.[2–4] However, aside from a limited number of studies, there has been little experimental validation of Co-60 conformal delivery.[5] Furthermore, while computed tomography imaging with Co-60 has been performed for basic medical physics studies,[6,7] there has been very little work done on the evaluation of the potential for image guidance with Co-60.

This report will review the results of experimental investigations of the potential of Co-60–based IGRT via tomotherapy — a rotational implement-ation of IMRT.[8,5] Measured conformal dose distributions achieved with an in-house Co-60 tomotherapy benchtop apparatus will be compared to the corresponding treatment plans. The results of investigations of Co-60 megavoltage computed tomography (MVCT) for image guidance[5,6] will also be shown. The findings of this work support the fact that there is ample potential for administering modern radiation therapy with a cobalt unit and encourage further investigations and development.

## Materials and Methods

**Figure 1:**
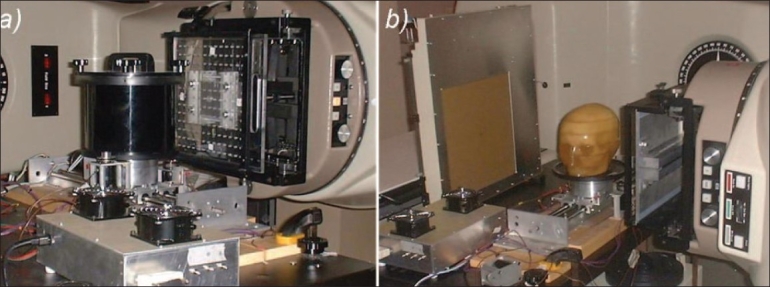
A photograph of the Theratronics T780 Co-60 unit incorporating the tomotherapy benchtop prototype for a) conformal irradiations on film and b) an imaging experiment with a Varian PortalVision LC250 EPID imager

All irradiations were performed on a Co-60 T780C unit (Best-Theratronics, Kanata, Canada) modified by the addition of a purpose-built computer-controlled rotate translate benchtop apparatus, as shown in Figure 1a. This first-generation tomotherapy test bed includes a rotate translate stage, which can move a phantom to be irradiated through a 1×1 cm^2^ Co-60 pencil beam. Intensity-modulated radiation therapy is performed in a single slice by varying the velocity of the phantom as it moves through the pencil beam. By this simple approach, we are well able to imitate the beam delivery from a NOMOS MIMiC multileaf collimator (BEST nomos, Pittsburg, PA, USA, formerly NOMOS Corp., Swickley, PA). It is possible to extend this delivery to 3D by adding a translate capability to the height of the table. Quantitative 3D delivery has been achieved in our laboratory, but the quantitative work reported here will be limited to two dimensions only. The test irradiations were performed on a cylindrical phantom containing GafChromic film (International Specialty Products, NJ, USA). Treatment planning was performed using an in-house developed inverse treatment planning system[9,10] that used either measured pencil beam data in an empirical Milan-Bentley–type algorithm[11] (simple phantom geometries) or EGSnrc/BEAMnrc[12] and EGSnrc/DOSXYZnrc[13] Monte Carlo simulations to model the beam delivery in patient CT data.

The in-house tomotherapy apparatus described above can also be used to perform fan-beam and cone-beam MVCT measurements. Our initial MVCT investigations were based on first-generation (using a pencil beam and diode detector with the image phantom translated through the beam to achieve one projection) and second-generation (using a fan beam and scanning diode) prototype Co-60 tomotherapy imaging systems. Recently our imaging studies have advanced with the development of a third-generation imaging system using a Varian PortalVision LC250 scanning liquid ionization chamber (SLIC) electronic portal imaging device (EPID) (Varian Medical Systems, Palo Alto, CA), shown in Figure 1b. In the Co-60 MVCT experiments, the phantom to be imaged was rotated on the central turntable; typically 180 and 451 image projections were taken through 360 degrees for the diode and SLIC experiments, respectively. The image reconstruction was based on parallel-beam filtered back-projection for diode-based imaging and a generalized Feldkamp technique[14] for EPID fan-beam imaging and was performed using in-house software written in Matlab (The Mathworks, Natick, MA) platform. Anthropomorphic head phantoms, a variety of orthopedic implants, and QA phantoms containing plugs of known electron density were used for qualitative and quantitative assessments.

## Results

**Figure 2:**
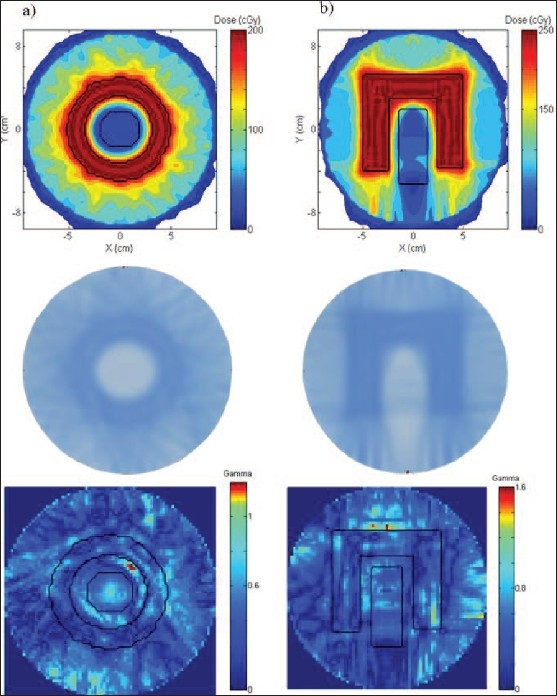
Cobalt-60–based tomotherapy plans of a “ring”-shaped structure (a) and an exaggerated concave “C” structure (b). Top: The dose distributions obtained with our in-house inverse treatment planning system; middle: the deliveries of both patterns on GafChromic film; bottom: gamma maps of the plans and deliveries using 5%, 5 mm gamma criteria

The findings to date confirm the viability of Co-60–based tomotherapy for conformal dose delivery. Figure 2 shows two examples of conformal irradiations planned for the delivery with the in-house cylindrical treatment phantom — a ring pattern [Figure 2a] and a standard conformal avoidance ‘C’ plan [Figure 2b]. These plans were implemented to determine whether the inverse treatment planning system would be able to generate an optimized and accurate dose distribution for simple but challenging geometry, including the ability to protect the central critical structure from unwanted radiation.

The ring in Figure 2a represents the PTV region and was prescribed an arbitrary dose of 200 cGy. The dose to the OAR (central structure) was limited to less than 60 cGy. The rest of the volume represents normal tissue (commonly known as the external region), and the dose to this region was restricted to less than 200 cGy. Using the in-house inverse treatment planning program based on aggressive active set (AAS) conjugate gradient method,[9] the plan was generated using 50 field orientations where each orientation used 31 beamlets to provide intensity modulation. The number of beamlets in our system represents the individual pencil beams being projected by the 31 leaf pairs of a multileaf collimator. Therefore, our configuration for field orientations and beamlets is similar to that typically used in commercial tomotherapy. The middle figure in Figure 2a shows the GafChromic film irradiation, which resembles the plan reasonably well. The bottom figure in Figure 2a shows a gamma map[15] of the simulated- and delivered-dose distributions. A gamma value less than or equal to 1 suggests that the agreement between delivery and simulation is within the specified criteria[15]: a standard clinical criterion allows a difference of up to 3% in dose and 3 mm in distance. The gamma comparison of the ring pattern showed that 87% of the treatment volume satisfied the 3%, 3 mm gamma criteria. In clinical practice, the goal is to have 95% of the volume satisfy the gamma criteria. Further analysis showed that 95% of the volume satisfies the gamma criteria if the criteria are 5% and 5 mm, and this is clearly illustrated by the gamma map shown in Figure 2a.

Figure 2b shows a similar plan of an exaggerated concave ‘C’ structure (the PTV) with an OAR at the center. This plan was generated using a similar number of beam orientations with a dose of 250 cGy to the PTV region and a maximum dose of 100 cGy to the OAR. The delivery was successful, as shown by the middle figure in Figure 2b. The gamma map analysis indicated that the plan and the delivery were in agreement with each other when using the gamma criteria of 5% dose difference and 5 mm distance difference and satisfied the 95% volume requirement.

Various treatment plans of similar complexity have been achieved and validated with the GafChromic film measurements.[16] These include simple conformal tests such as a star pattern, a circle, a ‘K’ pattern and some sophisticated experiments such as the common avoidance 'C' test and a simple head-and-neck test. All measurements showed good agreement with the corresponding plans, indicating that it is possible to deliver Co-60–based tomotherapy irradiations.

**Figure 3:**
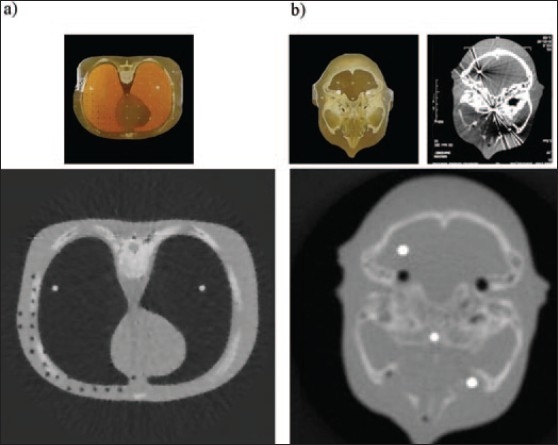
a) Photograph (top) and first-generation Co-60 CT image (bottom) of one torso slice of an anthropomorphic phantom; b) Photograph (top left), kVCT (top right) and third-generation Co-60 CT image (bottom) of one base-of-brain slice of the phantom

Imaging studies have been performed on a number of anthropomorphic phantoms using both a first-generation approach and a third-generation approach using the Varian PortalVision LC250 EPID imager [Figure 1b]. The resulting images illustrate that the Co-60 MVCT scanner performance is, 3 mm high-contrast spatial resolution and 2.8% low-contrast sensitivity (2.5-cm diameter objects). The image in Figure 3a shows one of the early torso images taken using our first-generation approach with a translated/rotated pencil beam and diode detector. Figure 3b shows a third-generation image of a head-and-neck phantom containing three stainless steel pins. The top right image in Figure 3b is of the same phantom obtained with kVCT and clearly shows the image-degrading artifacts associated with the metal pins. These artifacts are absent in the MVCT images shown below in Figure 3. Gross features prominent in the MVCT image include the occipital bone, posteria fosa, temporal bone, maxillary antrum, zygomatic bone, air hole for TLD, and nasal septum.

## Discussion

In this paper, we demonstrate that Cobalt-60–based tomotherapy approach is capable of both highly conformal intensity-modulated dose deliveries and viable image guidance. While it may seem that the addition of more complex beam collimation and megavoltage imaging to cobalt may complicate a simple technology, and perhaps make the device too complicated or expensive for worldwide distribution, we believe that it is important to establish full modern radiation therapy with a cobalt unit. It is quite clear that in the coming decade, the standard of care for radiation therapy will require image guidance and that radiation units incapable of imaging at the time of delivery will no longer be used in clinics. The development of tomotherapy type approaches with Co-60 also indicates that the potential for use of more sophisticated machines as proposed by Kron *et al.* incorporating MRI[17] may also translate into actual practice. We believe that there are considerable clinical and economic advantages in further investigating modern delivery with Co-60.

## Conclusions

Our experiments have confirmed the results of our previous modeling studies that Cobalt-60–based tomotherapy is clinically viable. Two-dimensional conformal dose delivery in a single slice with Co-60 has been validated experimentally using radiochromic film dosimetry. We have successfully developed Co-60 MVCT imaging techniques which provide adequate image quality required for IGRT. We believe that these results clearly indicate that undertaking the task of further development and implementation of modern radiation techniques based on Co-60 units is fully warranted.
